# The Structure, Activation and Signaling of IRE1 and Its Role in Determining Cell Fate

**DOI:** 10.3390/biomedicines9020156

**Published:** 2021-02-05

**Authors:** Natalia Siwecka, Wioletta Rozpędek-Kamińska, Adam Wawrzynkiewicz, Dariusz Pytel, J. Alan Diehl, Ireneusz Majsterek

**Affiliations:** 1 Department of Clinical Chemistry and Biochemistry, Medical University of Lodz, 90-419 Lodz, Poland; natalia.siwecka@stud.umed.lodz.pl (N.S.); wioletta.rozpedek@umed.lodz.pl (W.R.-K.); adam.wawrzynkiewicz@stud.umed.lodz.pl (A.W.); or pytel@musc.edu (D.P.); 2 Hollings Cancer Center, Department of Biochemistry and Molecular Biology, Medical University of South Carolina, Charleston, SC 29425, USA; jad283@case.edu; 3 Department of Biochemistry, School of Medicine, Case Western Reserve University, Cleveland, OH 44106, USA

**Keywords:** inositol-requiring enzyme type 1 (IRE1), endoplasmic reticulum (ER) stress, Unfolded Protein Response (UPR), factor X-box binding protein 1 (XBP1), Regulated IRE1-Dependent Decay (RIDD), c-Jun N-terminal kinase (JNK), apoptosis, drug development

## Abstract

Inositol-requiring enzyme type 1 (IRE1) is a serine/threonine kinase acting as one of three branches of the Unfolded Protein Response (UPR) signaling pathway, which is activated upon endoplasmic reticulum (ER) stress conditions. It is known to be capable of inducing both pro-survival and pro-apoptotic cellular responses, which are strictly related to numerous human pathologies. Among others, IRE1 activity has been confirmed to be increased in cancer, neurodegeneration, inflammatory and metabolic disorders, which are associated with an accumulation of misfolded proteins within ER lumen and the resulting ER stress conditions. Emerging evidence suggests that genetic or pharmacological modulation of IRE1 may have a significant impact on cell viability, and thus may be a promising step forward towards development of novel therapeutic strategies. In this review, we extensively describe the structural analysis of IRE1 molecule, the molecular dynamics associated with IRE1 activation, and interconnection between it and the other branches of the UPR with regard to its potential use as a therapeutic target. Detailed knowledge of the molecular characteristics of the IRE1 protein and its activation may allow the design of specific kinase or RNase modulators that may act as drug candidates.

## 1. Introduction

The Endoplasmic Reticulum (ER) constitutes the major membrane trafficking system responsible for the folding and assembly of most of the secretory proteins, as well as protein quality control [1,2]. Physiological or pathological stimuli generate stress in the ER through the accumulation of misfolded proteins [3]. Under such circumstances, a signaling network termed the Unfolded Protein Response (UPR) becomes activated in order to rebalance the ER homeostasis [4]. At the initial phase of the UPR, the adaptive response is triggered to relieve this stress. Upon activation of the above-mentioned pro-adaptive branch of the UPR, the synthesis of ER resident chaperones and folding catalysts occurs in order to increase the ER folding capacity, whereas the folding load in the ER is decreased through attenuation of the global mRNA translation and increased clearance of misfolded proteins by ER-associated degradation (ERAD). However, if the ER stress conditions are prolonged and severe, the pro-apoptotic branch of the UPR signaling pathway is activated [5].

The three ER-resident transmembrane sensors comprise inositol-requiring enzyme type 1 (IRE1), activating transcription factor 6 (ATF6), and protein kinase R (PKR)-like endoplasmic reticulum kinase (PERK). All these proteins become activated upon ER stress conditions and act as molecular gatekeepers of the UPR [6].

Among the UPR-related proteins, IRE1 is said to be the most evolutionarily conserved as it has been found not only in animals, but also in plants and the yeast *Saccharomyces cerevisiae* [7]. IRE1 consists of two domains: The N-terminal, ER luminal domain, which senses unfolded proteins, and C-terminal cytoplasmic region, which initiates UPR via serine/threonine kinase and endoribonuclease (RNase) domains [8]. The latter becomes activated via conformational change, autophosphorylation, and higher-order assembly [9]. Like PERK, IRE1 is capable of inducing cell fate by two distinct routes: The first by induction of adaptive cellular response by unconventional splicing of the transcription factor X-box binding protein 1 (XBP1) mRNA or Regulated IRE1-Dependent Decay (RIDD) posttranscriptional modifications, and the second by activation of the pro-apoptotic c-Jun N-terminal kinase (JNK), which takes place under prolonged or severe stress conditions [10,11].

**Figure 1:**
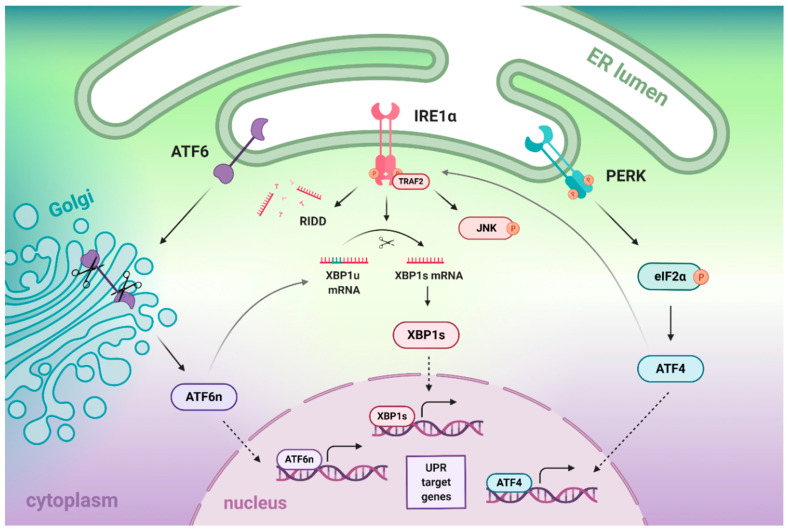
The structure of the Unfolded Protein Response (UPR)-dependent signaling branches and the crosstalk between them. Upon ER stress, the activating transcription factor 6 (ATF6) undergoes proteolytic cleavage in the Golgi apparatus, wherein its transcriptionally active form (ATF6n) is produced. The inositol-requiring enzyme type 1α (IRE1α) possesses several activities: It induces the regulated IRE1-Dependent Decay (RIDD) of specific mRNAs, the unconventional splicing of *x-box binding protein 1* (*XBP1)* mRNA, as well as it interacts with the TNF Receptor Associated Factor 2 (TRAF2) to initiate the c-Jun N-terminal kinase (JNK) signaling cascade. Activated protein kinase R (PKR)-like endoplasmic reticulum kinase (PERK) phosphorylates the eukaryotic initiation factor 2α (eIF2α), which blocks general translation initiation and selectively induces translation of particular mRNAs at the same time, including activating transcription factor 4 (ATF4). The downstream targets of all UPR branches, namely ATF6n, ATF4, and the spliced form of XBP1 (XBP1s), translocate to the nucleus to regulate the transcription of UPR target genes. Additionally, both ATF6n and ATF4 upregulate IRE1α activity, via induction of *XBP1* mRNA and increase in IRE1α expression, respectively.

The other two mammalian ER stress sensors of UPR signaling are PERK and ATF6; they possess similar structures to IRE1: An ER luminal stress-sensing domain, a transmembrane region, and a cytoplasmic enzymatic domain. Among all three transducers, PERK becomes activated first, followed by IRE1 and ATF6 [12]. Each of the transducers independently governs distinct branches of the UPR signal transduction pathways. When activated, PERK phosphorylates eukaryotic initiation factor 2α (eIF2α), which transiently inhibits general protein translation within the cell; this prevents further increases in protein-folding demand in the ER and inhibits cellular protein synthesis by reducing the load of proteins entering the ER [13,14]. Simultaneously, phosphorylated eIF2α selectively activates translation of particular mRNAs containing small open reading frames in their 59 untranslated regions, including activating transcription factor 4 (ATF4), which regulates the transcription of UPR target genes [15]. When stress conditions become prolonged or too severe to cope with, ATF4 upregulates the transcription of a gene encoding for pro-apoptotic C/EBP homologous protein (CHOP) [16]. By contrast, upon ER stress, ATF6 is being packed into transport vesicles and relocates from the ER to the Golgi apparatus where it undergoes proteolytic cleavage by the site 1 (S1P) and site 2 (S2P) proteases [17]. Next, the cleaved cytosolic domain of ATF6 (ATF6n) enters the nucleus, where it regulates the UPR target genes [5,18] (Figure 1).

Numerous studies have confirmed the upregulation or presence of activated forms of IRE1, implying it plays a role either in physiological processes or in the pathogenesis of numerous human diseases [19,20,21,22,23,24]. For instance, increased IRE1 activity has been confirmed in cancer, neurodegenerative, inflammatory, and metabolic disorders, which are strictly associated with accumulation of malfolded proteins and induction of ER stress [25,26,27,28]. These findings shed light on the potential of IRE1 as a novel target in personalized molecular therapies [29,30].

To date, several inhibitory compounds targeting IRE1 have been developed, yet little is currently known of their properties [31,32]. Moreover, the exact mechanisms regulating IRE1 activity and the way it resolves upon late ER stress have not yet been comprehensively described [33]. It is of utmost importance to understand how intensity and duration of the response to ER stress dictate the cell fate and thus to establish the balance between pro-survival and pro-apoptotic IRE1-dependent signaling [6]. Recent studies have described IRE1 as a master regulator in cell fate determination rather than a positive regulator for cell survival upon ER stress conditions [34,35]. The discovery of novel IRE1-dependent regulatory events indicates that, besides the protein-folding status, they appear to be essential for maintenance of cellular homeostasis within the cell [5]. This review aims to address mentioned issues and elucidate the recent groundbreaking findings in the field of research on IRE1-related molecular events.

## 2. The Crosstalk between IRE1α, ATF6, and PERK Branches of the UPR

### 2.1. Crosstalk between IRE1- and ATF6-Dependent Signaling Branches

The ATF6 branch of UPR, which becomes activated by intramembrane proteolysis, induces the *XBP1* mRNA before it undergoes the IRE1-dependent unconventional splicing. Importantly, the activated IRE1/XBP1 pathway is known to exert broader effects on ER stress-triggered transcription than ATF6 itself. However, significant levels of *XBP1* mRNA have to be induced before spliced form of XBP1 (XBP1s) is produced in amounts sufficient for its detection and transactivation [36]. In addition, IRE1α can splice a negligible amount of *XBP1* mRNA, if they were not generated beforehand by ATF6 [37]. Mentioned findings make ATF6 cleavage absolutely essential for IRE1α/XBP1-intitiated transcription of UPR target genes. Conversely, IRE1α is not required for the cleavage, nuclear translocation, or transcriptional activation of ATF6 [36]. The fact that the splicing by IRE1α increases the XBP1 potential to autoregulate its own transcription implies the requirement of both ATF6- and IRE1α-mediated processing for full UPR activation [38].

It is important to note that the active form of ATF6, which is derived from the preexisting precursor protein, is generated faster than XBP1s, which has to be newly translated from the previously induced and spliced mRNA. Oddly enough, ER stress does not drive the upregulation of *ATF6* mRNA. It has also been proposed that IRE1 and ATF6 activities have differing time courses: Low ER stress levels are dealt with by ATF6 activation only, medium-to-high levels of ER stress by activation of both ATF6 and IRE1/XBP1 axes, whilst extremely high levels are managed by multiple rounds of XBP1 cycle activation [36]. According to one model, ER stress first induces ATF6-driven upregulation of chaperones; however, in cases of persistent stress, IRE1 mediates production of ERAD components, such as ER degradation-enhancing α-mannosidase-like protein (EDEM) or E3 ubiquitin ligase HRD1, which promote degradation of misfolded proteins [39]. Another study has demonstrated that loss of ATF6 expression led to uncontrolled IRE1-reporter activity and resulting increase in XBP1 splicing upon ER stress, which implies that ER stress-mediated IRE1 signaling may possess an ATF6-dependent ‘off-switch’ [40]. Taken together, the utilization of the two transcriptional induction systems, IRE1- and ATF6-dependent, may constitute a fail-safe mechanism that enables the cell to cope with ER stress more effectively, regardless of the amounts of accumulation of unfolded proteins. Yet, the maximal activation of the UPR is achieved only when both systems are activated.

### 2.2. Crosstalk between IRE1- and PERK-Dependent Signaling Pathways

Recently, it has been reported that PERK/ATF4 arm of the UPR may also induce the IRE1α/XBP1 pathway in ER-stressed cell, in addition to ATF6: ATF4 upregulates the expression of *IRE1α* mRNA in HeLa cells and mouse embryonic fibroblasts (MEFs), which as a result increases the splicing ratio of XBP1 [37]. Thus, the output of the IRE1α/XBP1 pathway seems to be regulated separately by PERK/ATF4 and ATF6 so as to reinforce the cell against various types and intensities of stress. Intriguingly, differing effects of IRE1 splicing activity on ATF4 regulation were observed in distinct cellular models. For instance, genetic or pharmacological inhibition of XBP1 resulted in exacerbated ATF4/CHOP signaling in mouse oligodendrocyte progenitors and sensitized cells to ER stress [41]. In lung mucosal conventional dendritic cells type 1 (cDC1s), XBP1 deficiency resulted in execution of apoptosis, whilst intestinal cDC1s appeared to be quite resistant to XBP1 loss. This phenomenon was confirmed not to be CHOP- or JNK-dependent but was rather associated with modulation of RIDD activity and ATF4-mediated adaptive integrated stress response [10]. Mentioned findings are indicative not only of crosstalk between IRE1/XBP1 and PERK/ATF4 pathways, but also cell- and tissue-specific regulation of IRE1/XBP1 activity.

Mounting evidence suggests that the duration of PERK and IRE1 signaling significantly varies after the imposition of protein misfolding. For instance, it has been established that the IRE1 branch becomes inactivated upon irresolvable ER stress conditions, whilst PERK signaling remains unaffected and leads to cell apoptosis [42]. These results are in accordance with another study, in which activated IRE1 exerted a cytoprotective effect for its RNAse function in isogenic human cells. Consequently, selectively activated and persistent PERK signaling contributed to cell death upon chronic ER stress conditions, whereas the equivalent duration of IRE1 signaling did not [5].

In contrast to IRE1, which degrades *death receptor 5 (DR5)* mRNA via RIDD, PERK promotes cell death by activating *DR5* and other pro-apoptotic genes in a phosphatase RNA polymerase II-associated protein 2 (RPAP2)-dependent manner. It has been proposed that the mechanism of IRE1 attenuation by PERK/RPAP2 aims to abort failed adaptation to ER stress and trigger apoptosis [43]. In a separated experiment, the depletion of PERK inhibited RIDD in a substrate-specific manner, and this effect was fully restored by blocking the translation of the stem-loop (SL) region of target mRNAs, which is indicative of the involvement of PERK in the spatial and temporal regulation of RIDD [44].

In HEK293 cells, persistent ER stress conditions attenuated IRE1 and ATF6 activities with maintained PERK/CHOP signaling; however, artificially sustained IRE1 activity enhanced cell survival [42]. Consistently, early onset and attenuation of XBP1 splicing in the IRE1-reporter SH-SY5Y cells emerged as cytoprotective, whereas shRNA-dependent inhibition of IRE1/XBP1s expression resulted in early cell death. By contrast, silencing of PERK had no impact on overall rate of ER stress-induced cell death, but it markedly sensitized cells to ER stress shortly after treatment with the corresponding ER stressors [45]. Along these lines, it is the relative timing of IRE1 and PERK signaling that is responsible for the shift from cell survival to apoptosis, rather than preferential activation of a single UPR branch or switch from one branch to the other. There is strong evidence that early depletion of IRE1 in cells is lethal, and that the progression of the UPR towards the pro-apoptotic signaling depends on IRE1 signaling attenuation with the coexisting sustained PERK activity. To sum up, the duration of UPR signaling pathways seems to be a key factor in ER stress-related cell fate decisions.

### 2.3. Coronavirus-Mediated Activation of the UPR Signaling Pathways and Their Possible Crosstalk

In view of the current epidemiological situation with COVID-19 pandemic, multiple studies have aimed to establish the role of IRE1 signaling in the severe acute respiratory syndrome coronavirus (SARS-CoV) infections [46]. In study by Versteeg et al., SARS-CoV did not induce IRE1-dependent XBP1 splicing [47], as compared to the other known coronavirus, murine hepatitis virus (MHV) [47,48]. These results are in compliance with another interesting finding by Chan et al., who have confirmed that SARS-CoV spike (S) protein in fact accumulated in the ER and selectively induced activation of PERK/CHOP branch of the UPR with the simultaneous upregulation of GRP78/94 in 293FT cells, but it did not affect *XBP1* mRNA splicing nor ATF6 transcriptional activity [49]. However, it has recently been suggested that SARS-CoV-2 may affect IRE1 activity indirectly, as the Non-Structural Protein 6 (NSP6) in its membrane interacts with Sigma receptor 1, which is known to control the activation of IRE1 [50].

On the other hand, study on another coronavirus, infectious bronchitis virus (IBV), found that IBV-induced autophagy required activity of IRE1, but not of its substrate, XBP1 [51]. By contrast, IRE1α protected IBV-infected cells from apoptosis and this effect resulted from both kinase and RNase activity, and the subsequent conversion of XBP1 from its pro-apoptotic form, XBP1u, towards the pro-survival XBP1s protein; in addition, the IRE1α-dependent cytoprotection was found to involve modulation of JNK phosphorylation and the RAC-alpha serine/threonine-protein kinase (Akt) signaling [52]. As CoV requires formation of ER-derived vesicles for its replication cycle, with the subsequent RNA synthesis and assembly of virions out of viral structural proteins at the ER-Golgi intermediate compartment, it has been implied that SARS-CoV specifically modulates the UPR to facilitate the replication process [50].

## 3. The Structure of IRE1α and Molecular Events Associated with Its Activation

### 3.1. The Inactive and Active States of the IRE1 upon Distinct Molecular Events

ERdj4/DNAJB9, a J domain co-chaperone of immunoglobin-binding protein (BiP), has been identified to function as a selective IRE1α repressor that promotes the formation of a complex between the N-terminal luminal domain (NLD) of IRE1α and BiP. Upon association with NLD, ERdj4 recruits BiP by the induction of ATP hydrolysis, which forcibly disrupts the IRE1 dimers. The active, dimeric state of IRE1 can be subsequently restored by the unfolded proteins competing for BiP [53]. In accordance with this theory, BiP has been suggested to promote monomerization of IRE1 and thus initiate active repression of IRE1-dependent signaling upon waning ER stress, rather than play a role of a direct ER stress sensor [54]. Consistently, BiP buffers IRE1 activity via stabilization of its monomers in non-stressed conditions until they are activated by unfolded protein ligands [55]. As ERdj4 is specifically related to IRE1, and it does not regulate the activity of the other major UPR effectors like PERK, it would be an interesting target for the assessment of indirect modulation of the IRE1-dependent signaling.

Recently, an allosteric mechanism for UPR induction has been suggested instead of the previously described competition model; this theory is based either on qualitative pull-down assays or in vitro Förster resonance energy transfer (FRET) assay. In the proposed model, the interaction between IRE1 and BiP is mediated via the ATPase domain of BiP independently of nucleotides, which discounts a chaperone-substrate type interaction. When the misfolded protein becomes recognized or sensed by the BiP and binds to its substrate-binding domain (SBD), the allosteric changes occur resulting in the dissociation from IRE1, which does not involve ATPase activity. Thus, the binding to IRE1 and detection of misfolded proteins require two different domains of BiP coupled by conformational change [56].

According to the other theory, NLD can bind peptides and unfolded proteins with a characteristic amino acid bias to the MHC-like groove, which induces allosteric changes and leads to oligomerization. Consequently, specific mutations of a hydrophobic patch at the dimer interface have been shown to disturb binding and oligomerization process, and the impaired oligomerization of NLD led to abolishment of IRE1 activity in living cells [57]. However, in view of the presumptions that MHC-like groove is too narrow and not hydrophobic enough to accommodate a peptide, it cannot be excluded that other possibilities may exist, such as multiple binding sites within the IRE1 molecule [58]. It is likely that NLD may bind misfolded proteins via a conserved hydrophobic groove, in a manner similar to that of PERK [59]. Nevertheless, it has been demonstrated that besides direct binding of misfolded protein, the IRE1 activation depends primarily on dissociation of BiP [60].

**Figure 2:**
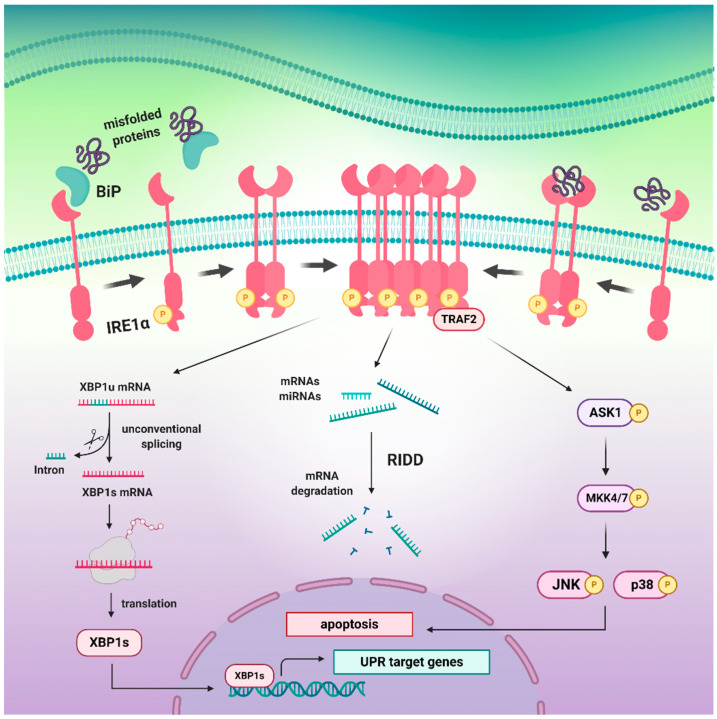
The activation of human inositol-requiring enzyme type 1α (IRE1α)-dependent signaling pathway with its downstream targets. The IRE1α activation occurs upon association of binding immunoglobulin protein (BiP) with misfolded protein and its subsequent dissociation from IRE1α, or alternatively upon direct binding of misfolded protein by the N-terminal luminal domain (NLD) of IRE1α. The following oligomerization, often of more than four IRE1α molecules, is required for further trans-autophosphorylation process and activation of kinase and RNase domains. The full kinase/RNase activity positively correlates with the increasing number of IRE1α oligomers. Activated IRE1α induces the following cellular processes: The unconventional splicing of *x-box binding protein 1* (*XBP1*) mRNA, regulated IRE1-Dependent Decay (RIDD) and TNF Receptor Associated Factor 2/apoptosis signal-regulating kinase 1/mitogen-activated protein kinase kinase4/7/c-Jun N-terminal kinase/p38 (TRAF2/ASK1/MKK4/7/JNK/p38) signaling pathway. The pro-survival or pro-apoptotic effect of IRE1α activation is exerted by selective induction of its specific branches and depends on numerous factors, mainly the severity and timing of cellular stress conditions.

Recent findings have also suggested that IRE may in fact be induced not only by the misfolded proteins, but also by the aberrant lipid compositions of the ER membrane, referred to as a lipid bilayer stress. It has been demonstrated that for this purpose, IRE1 uses an amphipathic helix (AH), which enables for sensing membrane aberrancies and control of UPR activity [61]. In view of the fact that PERK has been found to possess an intrinsic lipid kinase activity towards diacylglycerol (DAG) by which it generates phosphatidic acid (PA) and regulates Akt, mTOR, and mitogenic pathways [62], it would be worth investigation whether IRE1 exhibits similar activities (Figure 2).

One key regulator of IRE1 activity constitutes the ribonuclease inhibitor called ribonuclease/angiogenin inhibitor 1 (RNH1), a leucine-rich repeat domains-containing protein that binds to and inhibits the RNase domain of IRE1. That said, RNH1 is regarded as a direct negative controller of the IRE1 branch that attenuates IRE1-dependent signaling upon ER stress [6]. Ubiquitin-specific protease 14 (USP14), a protein that also interacts with the cytosolic portion of IRE1, is on the other hand regarded as physiological inhibitor of ERAD machinery under non-stressed conditions, and, conversely, is inhibited by ER stress [63]. The other modulator proteins known to directly interact with IRE1 include heat shock proteins (HSPs), poly (ADP-ribose) polymerase 16 (PARP16), RING finger protein 13 (RNF13), pro-apoptotic proteins Bax and Bak, and Bax inhibitor-1 (BI-1) [34].

Several pieces of evidence indicate the ability of IRE1 to autoregulate its activity. In this regard, Ser840, Ser841, Ser850, and Thr844 have been identified as the regulatory sites within the activation segment of the IRE1 [64]. Mechanistically, the self-association of IRE1 facilitates the activation loop phosphorylation, which unlocks the autoinhibition in the kinase domain, and the RNase activation induces the conformational rearrangement of IRE1 dimers from face-to-face to back-to-back [65]. The structural analysis revealed that in such back-to-back conformation, the kinase domain is primed for catalysis with the RNase domains being disengaged. It has also been suggested that IRE1 may in fact be autoinhibited in a Tyr-down mechanism, similar to the one found in the unrelated serine/threonine protein kinase Nek7, and that the kinase activity may be potently inhibited while the XBP1 splicing remains stimulated at the same time [66].

### 3.2. Details of the Structure of IRE1

Two IRE1 genes have been identified in the mammalian genome: IRE1α and IRE1β. The former is expressed in all types of cells and tissues, whereas the latter is expressed only in the intestinal epithelium [67]. The structure of both forms of IRE1 is similar to that of TGF-β serine/threonine protein kinase receptors and mechanistically they are similar to receptor tyrosine kinases, depending on ligand binding-induced dimerization as the activation mechanism for the cytoplasmic domains [68]. 

The N-terminal luminal domain (NLD) on the other hand, which serves as an ER stress sensor, appears to rely on the ligand-independent activation mechanism [69]. The crystal structure of the monomer of the NLD of human IRE1α has been found to comprise 367 amino acid residues (S24–V390) organized into a unique fold of triangular β-sheet cluster. Structural analysis revealed the three sides of the triangular plate to include three major β-structural motifs (namely N, C, and M), which are connected with several α-helices inserted between them [67]. Additionally, an 18 amino acid signal sequence (SS) is localized at the N-terminus [70].

The cytoplasmic region of IRE1 consists of two major parts: The kinase domain and the structurally continuous kinase-extension nuclease (KEN)/RNase domain localized at the C-end of the molecule. The protein kinase domain (residues 571–832) has a characteristic bilobal fold with the ATP-binding site located in the cleft between the β-sheet N-terminal lobe and the α-helical C-terminal lobe [64], and it comprises the C-helix (residues 603–623) and the activation segment (residues: 711–741) [71]. The central B10 residues (720–729) of the kinase activation segment are disordered and contain a potential phosphorylation site at Ser724, the disordered residues 746–748 carry the APE motif, residues 711–713―the DFG motif, whereas the RNase domain (residues 835–963) has a helical conformation with a fully ordered connection between helices 3 and 4 [72].

### 3.3. Structural Changes and the Different Activities of IRE1

Upon activation, the NLD forms stable dimers with a molecular mass of approximately 96 kDa, and the extensive dimerization interface is additionally stabilized by hydrogen bonds and hydrophobic interactions. Dimerization creates an MHC-like groove at the interface, which does not require peptide binding [67,73]. Under normal conditions, the NLD interacts with the ER resident chaperone, BiP, which maintains IRE1 in a monomeric state. Mechanistically, when misfolded proteins accumulate, BiP binds to the misfolded protein and dissociates from IRE1, allowing for dimerization of the released NLD to occur [69]. The association of the NLDs of two IRE1 molecules on the opposite sides of the ER membrane brings the cytoplasmic kinase domains into close proximity, which in turn drives transphosphorylation [57]. For this purpose, the kinase domains juxtapose in a face-to-face orientation, which allows for the binding of nucleotides such as ADP or ATP. Once ATP binds, the interaction is strengthened by the active sites of the opposite monomers which can access each other in trans orientation [64,74]. In addition, anti-parallel β-sheet comprising residues of the N-terminal end of the partially unwound C-helix is formed to bolster and support the projecting activation loop. Such conformation directs the conserved phosphorylation target Ser724 at the tip of the activation segment from one IRE1α molecule towards the opposite one in the ‘phosphoryl-transfer’ competent dimeric face-to-face complex [43,72], and this phenomenon is referred to as a reciprocal face-to-face transphosphorylation.

These molecular interactions resulting in oligomeric association, trans-autophosphorylation, and cofactor binding within the kinase domain are essential for the activation of the RNase domain and for its splicing activity [64,75]. Recent structural work has implied that the inactive, dephosphorylated IRE1α is able to bind nucleotides in the presence of Mg^2+^, and that its autophosphorylation is competitively inhibited by ADP. Upon activation, IRE1α turns into a competent protein kinase capable of phosphorylating a heterologous peptide substrate. It has been proven that the sequence-specific RNase activity by which IRE1 is able to initiate *XBP1* mRNA splicing is an additional helical domain, which is structurally continuous with and fused to the C-terminal lobe of the kinase domain. Thus, the XBP1-specific RNase activity depends on autophosphorylation, which could be permitted by ATP-competitive inhibitors like staurosporine and sunitinib [72]. Intriguingly, several pieces of evidence suggest that, instead of phosphotransfer reaction, it is the conformational change in the kinase domain that is essential for IRE1 activation, as the binding of ligands such as ATP mimetic 1NM-PP1 or IPA to the ATP-binding pocket within kinase domain is able to activate RNase directly [34]. Studies in yeast have also demonstrated that the kinase activity of IRE1 can be entirely bypassed, and that specific IRE1 mutants with inactivated kinase domain are still able to oligomerize and induce splicing reaction upon ER stress [55].

Notably, direct binding of unfolded protein by IRE1 is not required for the formation of homodimer of catalytically functional RNase domains and induction of RNase activities within the cytosolic domain [60]. It is the release of BiP that initiates the first stage of intramolecular autophosphorylation which is dimerization-independent; however, complete activation of the RNase function requires dimerization-dependent intermolecular autophosphorylation. Thus, the activation of IRE1α occurs in stages, each of which correlates with increasing degrees of autophosphorylation and resulting RNase activity [67,75]. Moreover, the newest findings have identified the five amino acid residues within IRE1α molecule, which are required for RNase activity but not kinase activity [76], which indicates that the kinase and RNase activities of IRE1 are in fact independent and that it is the RNase activity that is actually essential for UPR induction. The oligomerization of the kinase/RNase domain of IRE1 has been therefore established as an intrinsic mechanistic requirement for its full activity [77,78], and it has been suggested that preventing dimerization by phosphatase action or disrupting homodimer formation may constitute a target in terms of IRE1 inhibitor designing [76].

It has been demonstrated that upon activation, IRE1 forms oligomers larger than four molecules characterized by complex topologies and higher-order organization [79]. In another study, the oligomer of IRE1 cytosolic domains was obtained in a form of a rod-shaped assembly, which had no known precedence among kinases [77]. Such clusters remain anchored into the ER membrane, with bunches of IRE1 molecules being trapped within the assembly. When the ER stress attenuates, disassembly occurs, and the constituent molecules of IRE1 clusters are released back into the ER network in a distinct manner than that of cluster assemble. Whilst the actual role of IRE1 clusters remains elusive, it is speculated that they act as temporary storage compartments for hyperactivated IRE1 or excessive IRE1 molecules, behaving as some sort of buffering mechanism against overactivation of IRE1; alternatively, they may target different mRNA substrates than single IRE1 subunits, with the former ones selectively inducing XBP1 mRNA processing and the latter―RIDD [9,80]. This is in accordance with the recent finding that IRE1 clustering and RNase activity are in fact functionally independent of each other. Although both processes occur upon ER stress conditions within similar timeframes, RNase-inactive IRE1 molecules are still capable of assembly, and clustering is not necessary for full RNase activity. Interestingly, such clusters of RNase-inactive IRE1 are characterized by different morphology and dynamics than the active ones [81].

**Figure 3:**
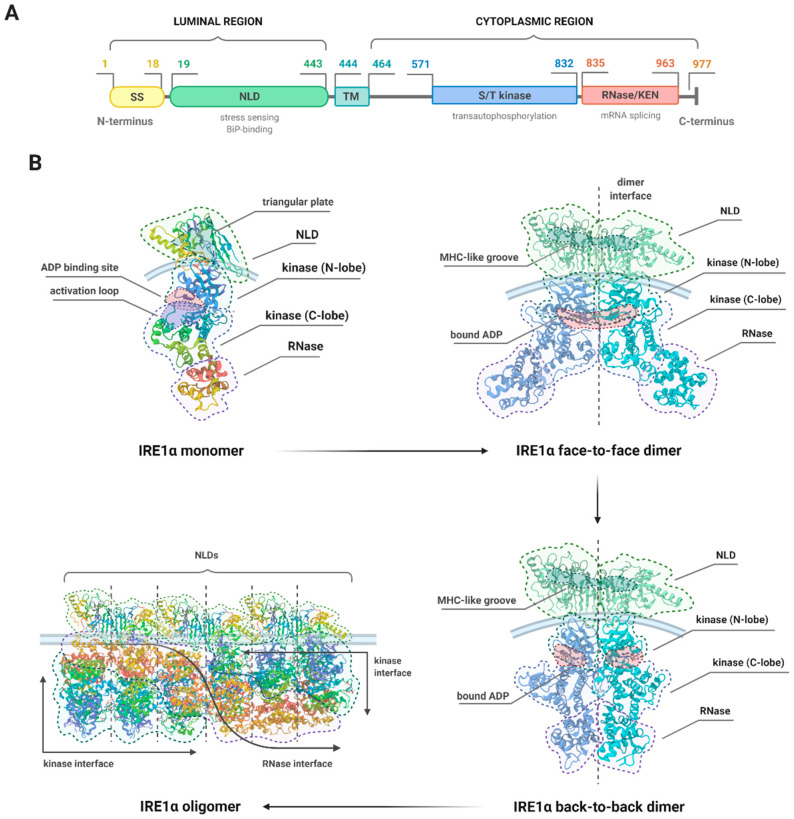
The molecular insight into the structure of human inositol-requiring enzyme type 1α (IRE1α) (**A**) and the structural changes occurring upon its activation (**B**). (**A**) At the N-terminal end of IRE1α molecule, there is an 18 amino acid signal sequence (SS) and the N-terminal luminal domain (NLD), consisting of three major β-sheets forming a triangular plate. Either the transmembrane region (TM) or the serine/threonine kinase (S/T kinase) and endoribonuclease (RNase) domains of the cytoplasmic region consist mostly of α-helices, and the RNase domain extends to the C-terminus of IRE1α protein. (**B**) Upon activation, the luminal domains oligomerize, bringing the cytosolic kinase domains into juxtaposition, thus allowing for its trans-autophosphorylation and cofactor binding. This results in the activation of protein kinase, conformational change from “face-to-face” to “back-to-back”, and the subsequent induction of RNase activities within the cytoplasmic region. Activated IRE1α dimers further oligomerize to form a complex structure of more than four molecules, in a characteristic rod-shaped assembly with the extensive RNase surface. PDB ID codes for the used models: NLD: 2HZ6; cytoplasmic region: 4Z7G (monomer), 3P23 (face-to-face dimer), 2RIO (back-to-back dimer), 3FBV (oligomer).

Prolonged or unmitigated ER stress attenuates IRE1 signaling and makes it shift to a refractive state via dissolution of oligomers, dephosphorylation, and decline in RNase activity [82]. The newest findings not only suggest that the timing appears to be major regulator that governs IRE1 activity, but also that IRE1 signaling can be reset upon resolution of ER stress, which makes it in fact reversible. In general, three states of IRE1 activation have been described: An inactive state, an active state, and a refractive state in which IRE1 no longer responds to unresolved ER stress. It has also been suggested that the dephosphorylation process is pivotal for regulation of IRE1 activity and entering the refractive state, as anti-p-IRE1 antibodies recognize neither inactive nor refractive state [79] (Figure 3).

### 3.4. The Pro-Survival XBP1 Pathway Restores Cellular Proteostasis

After detection of elevated levels of malfolded proteins in the ER, IRE1 transmits a signal to the nucleus via spliceosome-independent splicing of mRNAs, which encode for bZIP transcription factors HAC1/XBP1 [76]. HAC1 serves as the master transcriptional regulator of the UPR in yeast, and XBP1 in mammals. Briefly, XBP1 pre-messenger RNA (pre-mRNA) is converted to mature mRNA in order to gain transcriptional activity. For the unconventional splicing, the interconnected activity of kinase and RNase domains is required to cleave mRNAs at the non-canonical splice sites before the translation occurs [8]. 

The IRE1-dependent removal of 26-nucleotide intron from XBP1 pre-mRNA with the subsequent re-ligation by RtcB introduces a translational frame shift through switching of the reading frame in the C-terminal portion of the respective proteins [64,83]. Besides the frame shift, activated IRE1 mediates the insertion of a new carboxyl domain in the XBP1 protein, so that the functional UPR activator, XBP1s, is eventually obtained [36]. XBP1s contains the nuclear localization signal and the transcriptional activation domain which induces transcription of target gene products required for the re-establishment of proteostasis, such as chaperones and other ER-resident proteins [18,84]. In contrast to RIDD, maximum XBP1 splicing activity requires the combined action by the subunits within the IRE1 oligomer [80].

Notably, XBP1 pre-mRNA is also translated into a functional protein XBP1u, a negative regulator of the UPR that accumulates upon the recovery phase of ER stress. This mechanism enables the cell to quickly adapt to environmental changes via switch of the proteins encoded in the XBP1 mRNA from a negative regulator to an activator [83,85,86]. Generally, the splicing reaction requires ongoing cellular transcription, whilst it does not require active translation nor nuclear processing of the RNA substrate, and that the dimerization of IRE1α is sufficient to initiate the splicing in the absence of the UPR activation [87]. It has been well known that the conventional splicing exclusively takes place in the nucleus, while the unconventional splicing of XBP1 mRNA occurs mainly in the cytoplasm [85,87,88]. However, as it has recently been indicated that the unconventional splicing may also take place in the nucleus, depending on the secondary structure of XBP1 mRNA [89], the actual mechanisms of this molecular event require further explanation.

### 3.5. Dual Role of RIDD Pathway in Cell Fate Regulation

Apart from the unconventional XBP1 mRNA splicing, IRE1 promotes the cleavage of mRNAs encoding for mostly ER-targeted proteins in the process called RIDD, which aims to reduce the cargo protein load in the ER during severe stress conditions. RIDD-mediated cleavage of RNA occurs at an XBP1-like consensus site, but in a manner divergent from XBP1 mRNA splicing. RIDD pathway involves the relatively promiscuous degradation of wide variety of membrane-associated mRNAs, which enables their rapid turnover, and the whole process requires the activity of IRE1 nuclease [90] as well as cellular exoribonucleases, e.g., exosomes. The mRNAs targeted by RIDD encode for proteins localized in the cytosol, nucleus, ER, or the secreted ones [91], and are sequence-specific; they possess a cleavage site with a consensus sequence (CTGCAG) and a secondary structure similar to that of SL of XBP1 mRNA [92]. Such XBP1-like SLs within the target mRNAs are necessary for RIDD operation [44].

Basal activity of RIDD is required to maintain ER homeostasis and it has been demonstrated to play key role in numerous physiological processes, such as endocrine pancreas and liver function, as well as functioning of other tissues that undergo intense secretory processes [93]. Intriguingly, in contrast to XBP1 mRNA splicing, which is regarded mainly as cytoprotective, RIDD has revealed many unexpected features and is capable of either preservation of ER homeostasis or induction of cell death [91]. According to the current knowledge, the selective activation of RIDD by IRE1 results in cell death, whilst XBP1s promotes cell survival. Induction of RIDD does not require oligomerization of active IRE1 subunits and it involves the same catalytic residues as unconventional splicing, but different substrate binding sites [80]. It has been proven that IRE1α is increased and activated, and the RIDD activity is intensified upon XBP1 depletion. Conversely, IRE1α is down-regulated to suppress apoptosis-inducible RIDD activity at low XBP1 mRNA expression, which is indicative of the importance of IRE1α protein/XBP1 mRNA ratio in biological processes [37].

It is believed that basal RIDD constitutes the first IRE1-induced homeostatic response to ER stress―if this mechanism is insufficient, XBP1 splicing is activated and RIDD increases; however, when ER stress is unmitigated, XBP1 mRNA splicing decreases, whereas RIDD ultimately initiates apoptosis. The action of pro-death RIDD depends on degradation of antiapoptotic pre-miRNAs, mainly anti-Casp2 pre-miRNAs, and mRNAs encoding pro-survival proteins [91]. The phosphotransfer function of IRE1 is not required for XBP1 splicing and upregulation of caspase-2 (Casp2) expression, but it is essential for the subsequent Casp2 cleavage and resulting initiation of apoptosis [94]. On the contrary, the pro-survival role of RIDD has been shown to depend on inhibition of certain proapoptotic proteins, including TNF-related apoptosis-inducing ligand (TRAIL) receptor 2 [95].

### 3.6. The IRE1α/TRAF2/JNK Arm Initiates the Pro-Apoptotic Signals

JNK constitutes the member of a mitogen-activated protein kinase (MAPK) family, and it emerges as a part of late ER stress response that promotes apoptosis. The JNK-dependent apoptotic pathway of the UPR becomes activated by the cytoplasmic kinase part of IRE1, which binds to the E3 ubiquitin ligase TRAF2, an adaptor protein coupling plasma membrane receptors, and one of the MAP3Ks, Apoptotic-Signaling Kinase-1 (ASK1/MAP3K5), with the formation of a complex. This in turn stimulates activation of ASK1 downstream targets, JNK/MAPK8/SAPK1 and p38 MAPK, which constitute the stress kinases that promote apoptosis. In general, the entire pathway of JNK activation, initiated in the ER by intracellular signals, resembles the one induced by the cell surface receptors in response to extracellular stimuli [96]. Not only does the JNK mediate apoptotic pathways, but it can also induce necrosis under certain ER stress conditions [97].

Upon activation, JNK phosphorylates its substrates Bcl-2 and Bim, with the former one being inhibited, and the latter activated, which contributes to initiation of the apoptotic cascade. On the other hand, p38 MAPK phosphorylates and activates CHOP, which favors apoptosis via upregulation of Bim and DR5 and simultaneous downregulation of Bcl-2. JNK-mediated phosphorylation causes Bcl-2 to lose its anti-apoptotic abilities as it fails to bind to pro-apoptotic BH3-only members of Bcl-2 family, as well as ER-to-mitochondria Ca^2+^ influx becomes induced in JNK-dependent manner. Conversely, the pro-apoptotic proteins Bax and Bak, which antagonize the ER Ca^2+^-modulating activities of Bcl-2 and Bcl-XL at the ER membrane, have been shown to bind to IRE1α, resulting in its pathological, pro-apoptotic activation [98]. When ER stress is triggered, the IRE1 branch prevents Bax- and Bak-mediated ER membrane permeabilization and the resulting cell death. This makes IRE1-deficient cells susceptible to leakage of ER contents, accumulation of calcium in the mitochondria, oxidative stress, and ultimately cell death [99].

IRE1α may promote apoptosis via both its protein kinase and RNase domains. For the establishment of a time window to adapt to ER stress conditions and avoid apoptosis, IRE1α induces RIDD-dependent degradation of *DR5* mRNA. On the other hand, the cleavage of *miRNA-17*, *-34a*, *-96,* and *-125b* are induced by the RNase domain to stabilize and promote translation of *TXNIP;* TXNIP orchestrates apoptosis in caspase-1- and interleukin 1β-dependent manner, and also involves *caspase-2* mRNAs. Besides, the sequestration of TRAF2 by IRE1α may contribute to the direct activation of caspase-12. However, the recent study has confirmed that the IRE1/TRAF2-dependent JNK activation in fact precedes the activation of XBP1, and that it involves the expression of several antiapoptotic genes, namely *cIap1/Birc2*, *cIap2/Birc3*, *Xiap,* and *Birc6.* Thus, it can be concluded that the JNK-dependent transcriptional induction of these targets contributes to the inhibition of apoptosis at the early stage of response to ER stress. It has also been suggested that JNK signaling becomes pro-apoptotic 12 h or later after ER stress induction [100].

More recently, a novel mechanism of caspase-mediated IRE1 cleavage has been detected in several leukemic cell lines. The cleavage occurred within the cytoplasmic linker region of IRE1, which separates the luminal and cytoplasmic moieties, and this resulted in disruption of IRE1-dependent signaling upon ER stress. Ectopic expression of the cleavage-generated fragment (LDTM), consisting of NLD and transmembrane region of IRE1, was shown to promote growth of multiple myeloma (MM) cells in vivo. Mechanistically, LDTM inhibited the recruitment of Bax to the mitochondria, so as to counteract the apoptotic signals [35].

## 4. Pharmacological Modulation of IRE1 Activity

In general, IRE1 inhibitors may be divided into specific subgroups: Type I and II kinase inhibitors that target the ATP-binding kinase domain, and RNase inhibitors that inhibit the RNase domain. The kinase inhibitors act by stabilizing kinase active sites of IRE1 in opposing conformations via interaction with the same ATP-binding pocket, which can selectively inhibit or activate various enzymatic potencies. Such binding provides a shift between the two conserved kinase conformations, C-helix in (active) and C-helix out (inactive) [55]. For instance, type II inhibitors are known to selectively stabilize the inactive conformation of the ATP-binding site, characterized by the outward movement of catalytically important DFG motif (so-called DFG-out conformation). Under such a DFG-out conformation, they are able to block both autophosphorylation and RNase activity by making contact with both the ATP-binding site and an adjacent allosteric site. They have also been demonstrated to inhibit the other IRE1 phosphorylation-dependent molecular events, like TRAF2 binding. The type I inhibitors, on the other hand, also interact with the ATP-binding site but do not penetrate the allosteric pocket and do not significantly affect RNase activity [101]. Thus, the kinase conformational state evidently has a crucial impact on inhibitor potency and selectivity, which should be taken into consideration in terms of kinase inhibitor drug discovery. Type II kinase inhibitors are generally considered to be more selective than type I inhibitors, although the underlying mechanism is poorly understood. Overall, kinase inhibitors constitute the nucleotide analogs or other ATP competitive ligands. Type I inhibitors of kinase domain include, among others, APY29, AT9283, AP26113, TAE684, AZD7762, VX-680, sunitinib, dasatinib and IPA [74,101,102], and the type II inhibitors: 1-(4-(8-amino-3-isopropylimidazo[1,5-a]pyrazin-1-yl)naphthalen-1-yl)-3-(3-(trifluoromethyl)phenyl)urea (C_27_H_23_F_3_N_6_O), and the series of kinase-inhibiting RNase attenuators (KIRAs) [74,103].

Recently, particular attention has been drawn towards IRE1-derived peptide fragments that selectively affect oligomerization and RNase activation, instead of the widely used pharmacological inhibitors. Such fragments align well in the ATP-binding pocket and are therefore characterized by high selectivity for IRE1 binding. This approach may also reduce off-target effects, as demonstrated by the inhibitors above: These peptides are structurally homologous and complementary to the IRE1 regions itself. By means of a structural homology approach, the development of 18–50 amino acid peptide fragments derived from the IRE1 cytosolic domain led to the identification of non-peptide substitutes out of the FDA-approved drugs, namely methotrexate, folinic acid, cefoperazone, and fludarabine phosphate. Furthermore, experiments performed in primary RADH87 and common U87 glioblastoma multiforme (GBM) cell lines have shown that all drugs influence IRE1 activity, with the IC50 values ranging between 0.2–2 μM, and thus they have similar potency to that of specific RNase inhibitor MKC-8866. More importantly, administration of the inhibitors sensitized GBM cells to chemotherapy with temozolomide (TMZ) [32]. This finding indicates for potential clinical relevance of the tested substances and the need for further exploration of their effectiveness against GBM in the other models. As these drugs are in widespread clinical use, their application as selective IRE1 inhibitors could overcome a plethora of peptide-related issues such as decreased bioavailability, instability, sheer size, and crossing the blood–brain barrier (BBB).

**Figure 4:**
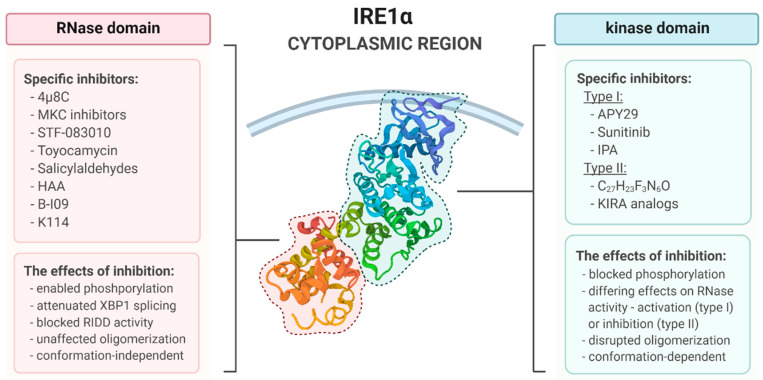
Inhibition of the respective domains within the cytosolic region of inositol-requiring enzyme type 1α (IRE1α) with the specific inhibitors and the major molecular implications associated with their utility.

Large-scale screening efforts have led to the discovery of the direct inhibitors of the IRE1 RNase domain like salicylaldehydes, 4μ8c, toyocamycin, series of MKC compounds, and STF-083110. Most of them constitute small hydrophobic molecules with the ortho-hydroxyl aryl aldehyde moiety; this forms a Schiff base with the K907 residue of IRE1 and allows for immersion within the hydrophobic pocket of the RNase domain [32,104]. Likewise, type II kinase inhibitors, such molecules indiscriminately shut off either XBP1 mRNA splicing or RIDD, providing a complete inhibition of distinct IRE1 functionalities [105]. Inhibition of RNase domain seems to be more clinically significant, as it demonstrates more predictable effects than targeting the kinase domain and does not exhibit as many off-target activities. Nevertheless, the application of specific IRE1 modulators has proven to be useful in the certain cellular contexts, as it allows the investigation of molecular events associated with selective activation or inhibition of the respective IRE1 activities. It may also clarify the exact role of IRE1-dependent signaling pathways in diverse physiological and pathological settings (Figure 4).

Importantly, despite numerous reports on therapeutic potential of activation of IRE1/XBP1-dependent signaling pathway and the cytoprotective effects that it exerts in certain pathological conditions, the subset of selective IRE1 RNase activators has not yet been extensively evaluated.

### 4.1. The Kinase Type I Inhbitors

#### 4.1.1. APY29

APY29 is an ATP-competitive, type I kinase inhibitor of IRE1α activity. It acts through interaction with an ATP-binding site, stabilization of kinase conformation, and allosteric-dependent activation of the adjacent RNase domain, even in the absence of ER stress. It has been shown to exert opposing effects on RNase activity to the type II inhibitor C_27_H_23_F_3_N_6_O, and it is also able to reverse its action. Structurally, the kinase catalytic domain of yeast IRE1 bound with APY29 is, as in the case of ATP or ADP, in an active conformation (DFG-in) [103]. Although APY29 is widely used in structural studies on IRE1 activity and drug development, it has been found to be toxic at low micromolar concentrations, which considerably limits its application in different cellular models [102].

#### 4.1.2. Sunitinib

Sunitinib is an FDA-approved anti-cancer drug that blocks the autophosphorylation activity of IRE1α in a dose-dependent manner and, as a type I pharmacophore, is regarded as a potent activator of IRE1 RNase activity. As in the case of APY29, sunitinib stabilizes an ATP-binding site conformation, which induces activation of the RNase domain [103]. The underlying mechanism of partial activation of the enzyme could be explained by the fact that the inhibitor fills the ATP-binding site, but not the phosphate or ribose subsites [77]. Unexpectedly, Sunitinib was demonstrated to effectively inhibit both IRE1 autophosphorylation and XBP1 mRNA splicing in H929 and U266 myeloma cells treated with tunicamycin (Tm) in vitro and in vivo. Thus, it was suggested that Sunitinib-mediated block of XBP1 splicing is a consequence of the autophosphorylation-dependent inhibition of IRE1 RNase activity of IRE1 in myeloma cells [72]. Sunitinib co-administered with chloroquine or/and gemcitabine exerted synergistic effect against pancreatic ductal adenocarcinoma (PDAC) in vitro and in vivo. Mice treated with all three compounds demonstrated increased survival, with the corresponding reduced BiP expression, decreased cell proliferation, and increased apoptosis in the pancreas [106]. Nonetheless, the contradictory effects that Sunitinib exerts on RNase domain in distinct experimental models require further exploration to establish its actual mechanism of action.

#### 4.1.3. IPA

IPA, a potent second-generation IRE1 activator of low molecular weight, activates the RNase domain via interaction with ATP-binding site, which in turn predisposes the kinase domain to oligomerize. A study in HEK293T cells revealed that IPA may in fact bind to PERK kinase active site and inhibit it or, at the low concentrations, activate a small subset of PERK molecules (15%) in a manner dependent on ligand-induced conformational changes [107]. Additionally, IPA exerted toxicity at nanomolar concentrations in treated cells, which presumably resulted from off-target binding of IPA to the other kinases. Mentioned findings indicate that IPA is not recommended to be used as a selective IRE1 modulator in terms of further research on cellular and organismal models [102].

### 4.2. The Kinase Type II Inhbitors

#### 4.2.1. C_27_H_23_F_3_N_6_O

C_27_H_23_F_3_N_6_O belongs to the second class of kinase inhibitors, which can inhibit RNase activity via interaction with the ATP-binding site, also under ER stress conditions. This is achieved by stabilization of kinase domain in a conformation alternative to that induced by first class inhibitors, so as to disable RNase activity. The above-mentioned inhibitor was able to suppress XBP1 mRNA splicing to a similar extent as a direct small molecule RNase inhibitor, STF-083010, and it also reduced autophosphorylation of IRE1α kinase domain in vitro in a dose-dependent manner, similarly to APY29. In the competition experiments, increasing concentrations of APY29 or sunitinib progressively reversed the inhibitory effect on RNase caused by C_27_H_23_F_3_N_6_O, and conversely, increasing concentrations of C_27_H_23_F_3_N_6_O inhibited RNase activity previously induced by APY29. This suggests that all tested inhibitors act on the same binding site, and that APY29 serves as a slight activator of RNase function [103].

#### 4.2.2. KIRA Analogs

In contrast to type I inhibitors, kinase inhibiting RNase attenuators (KIRAs) constitute ATP-competitive, imidazopyrazine-based inhibitors that inhibit RNase activity by allosteric interaction with the kinase domain. Structural analysis of 1-32 KIRA analogs revealed that these inhibitory compounds contain an aryl-urea moiety that stabilizes the kinase domain in a DFG-out inactive conformation [74]. Moreover, KIRAs have been demonstrated to specifically target distinct IRE1 oligomerization states and have the ability to break down IRE1 oligomers. KIRAs therefore preferentially block terminal RIDD activity triggered by hyperactive IRE1 levels over cytoprotective XBP1s signaling, and the effect is apparently dose-dependent [105]. Indeed, it has been found that a C_32_H_31_ClN_6_O_3_S KIRA inhibitor developed by Amgen projects an arylsulfonamide moiety, which causes the displacement of C-helix from the active conformation. This in turn disrupts the RNase interface of the active IRE1 back-to-back dimers, resulting in monomerization and loss of RNase activity [74]. The other report based on structural analyses proposed a model in which KIRAs act at an early stage of IRE1 activation via interaction with IRE1 face-to-face dimer formation, which as a result disables the RNase activation [71]. It has recently been discovered that KIRAs block the enzymatic activity not only of IRE1α, but also of the IRE1β isoform [108].

One of the KIRA analogs, KIRA6, has been tested in several pathological backgrounds with promising results―it preserved photoreceptor functional viability in ER stress-induced retinal degeneration rat model, function of pancreatic β cells in Akita diabetic mice, and the viability of Zika virus (ZIKV)-infected cells, but demonstrated no significant off-target effects after systemic administration [105,109,110]. Furthermore, in 5TGM1 cells, KIRA6 managed to suppress the bacterial subtilase cytotoxin (SubAB)-induced S729 phosphorylation of IRE1, whereas other inhibitors like staurosporine, imatinib, or sumatinib did not. KIRA6 had negligible impact on the XBP1s levels, and it also did not inhibit IRE1 phosphorylation at other sites [111]. However, in addition to IRE1, KIRA6 has recently been demonstrated to directly bind to KIT kinase at sub-micromolar concentrations and inhibit it; it was also found to induce cell death in a mast-cell leukemia cell line in a KIT-dependent manner [112]. In view of the mentioned off-target kinase inhibitory effects, the application of KIRA6 is limited for further research and instead, other selective IRE1 inhibitors should be applied. However, KIRA7 and KIRA8 analogs efficaciously mitigated ER stress-induced apoptosis in murine alveolar epithelial cells exposed to bleomycin. Both inhibitors prevented or even reversed pulmonary fibrosis in vivo, which makes them promising candidates in terms of anti-fibrotic drug development [113].

C_32_H_31_ClN_6_O_3_S and C_31_H_29_ClN_6_O_3_S (KIRA8), which were obtained by means of a high-throughput screen (HTS) of the Amgen library and structure–activity relationship (SAR) studies, proved to be inefficient in cellular models, despite having high selectivity for IRE1. Strikingly, screening of more than 300 native tumor cell lines against the investigated compounds did not reveal any effects on cell viability. Importantly, the panel included 15 MM cell lines, which are known to exhibit high levels of IRE1/XBP1s activity. These results contradict previous findings, implying that IRE1 does not play a critical role in tumor cell survival in vitro, and they call into question the utility of IRE1 inhibitors as novel antineoplastic agents [114]. Interestingly, further studies on KIRA8 revealed that it markedly inhibited the growth of MM and B-derived, nonmyeloma cancer cell lines in 3D cultures, while it had a much weaker impact on cells growing in 2D cultures. Moreover, the inhibitor managed to attenuate the growth of human MM xenografts in mice, either in subcutaneous (s.c.) and more clinically relevant orthometastatic models. Besides the antitumor effect, KIRA8 spared the function of nonmalignant hematopoietic cells, including plasma cells, primary hepatocytes, and pancreatic microislets, wherein it preserved insulin secretion in vitro; it was also well tolerated by treated animals [115].

### 4.3. RNase Inhbitors

#### 4.3.1. Salicylaldehydes

Salicylaldehyde analogs, which were identified through HTS, inhibit the site-specific cleavage of several mini-XBP1 SL RNAs as the substrates of IRE1 RNase in a dose-dependent manner. One potent salicylaldehyde analog is 3-ethoxy-5,6-dibromosalicylaldehyde, which has been demonstrated to bind to IRE1 in a specific, reversible, and dose-dependent fashion. Further in vitro analyses revealed that salicylaldehydes not only inhibit pharmacologically induced XBP1 mRNA processing, but they also block transcriptional upregulation of XBP1 target genes and RIDD-targeted mRNAs. Additionally, 3-methoxy-6-bromosalicylaldehyde effectively inhibited splicing of XBP1 mRNA in vivo, in Tm-treated mice [116]. The effectiveness of 3-methoxy-6-bromosalicylaldehyde has also been tested in several pancreatic cancer cell lines: In contrast to 2-hydroxy-1-naphthaldehyde (HNA), 3-methoxy-6-bromosalicylaldehyde inhibited the splicing of XBP1 mRNA, the colony formation in vitro, and it reduced tumor size in a mouse xenograft model [117].

#### 4.3.2. 4μ8C

Mechanistically, 4μ8C, which selectively binds to K907 in the RNase catalytic pocket with a formation of stable imine, blocks substrate access to the active site of IRE1 and consequently inhibits either XBP1 splicing or RIDD [118]. Treatment of H4IIE hepatoma cells with 4μ8C decreased splicing of XBP1 mRNA and RIDD activities of IRE1, regardless of presence of ER stress, and the drug significantly reduced cell proliferation in a dose-response manner. Notably, only higher concentrations of the inhibitor (60 μM) induced significant off-target effects [119]. 4μ8C effectively suppressed proliferation of luminal breast cancer cells and the aggressive phenotypes overexpressing IRE1, although the mechanism did not involve inhibition of XBP1 splicing [120]. By contrast, 4μ8C inhibited the XBP1 mRNA splicing in response to ER stress triggered by mutant proinsulin (C96Y) production in insulinoma cell line; however, the inhibition affected neither ERAD nor ER stress-induced apoptosis [121]. 4µ8C has also been shown to suppress inflammation in the arthritis mouse model and to counteract lipid-induced inflammation in in vitro and in vivo models of atherosclerotic disease [31]. Despite having remarkable selectivity for IRE1 in vitro, 4μ8C attenuated tumor growth, but did not evoke severe cytotoxicity in MM cell lines, whilst the inhibitor affected ER expansion and amylase secretion in AR42J cells co-treated with dexamethasone [118]. However, 4µ8C has demonstrated several off-target activities, as it decreased xanthine/xanthine oxidase- and angiotensin II-mediated ROS generation in vitro at low micromolar concentrations. Thus, it has been concluded that 4µ8C may act as a potent reactive oxygen species (ROS) scavenger and should be carefully used in research aimed at IRE1α inhibition [122]. Furthermore, the utility of 4μ8C in animal models seems to be limited due to its unfavorable pharmacokinetics [118].

#### 4.3.3. STF-083010

STF-083010, an RNase inhibitor, comprises an aldehyde moiety and an imine bond that undergoes hydrolysis in aqueous solution with a formation of an active product, 2-hydroxy-1-naphthaldehyde (HNA) [104]. HNA is smaller than STF-083010 but demonstrates full inhibitory potency [123]. Both inhibitory molecules have proven to be effective against several pancreatic cancer and chronic lymphocytic leukemia (CLL) cell lines, wherein they inhibited the XBP1 splicing reaction in a dose-dependent manner. Moreover, the normalized isobologram analysis revealed the synergistic effect of STF combined with bortezomib in four pancreatic cancer cell lines [117]. In acute myeloid leukemia (AML) cell lines and patient samples, either STF-083010 or HNA attenuated XBP1 mRNA splicing and evoked a cytotoxic effect. The apoptosis induced by inhibitors was caspase-dependent and involved G1 cell cycle arrest. HNA combined with bortezomib or As_2_O_3_ induced synergistic, cytotoxic effect against NB4 cells and AML patient-derived cells, depending on an increase in p-JNK levels and decrease in p-PI3K and p-MAPK levels. By contrast, normal human marrow mononuclear cells appear to remain unaffected upon treatment with HNA. As compared to STF-083010 and MKC-3946, HNA showed slightly greater inhibition of IRE1/XBP1s signaling [124]. In patient-derived pre-B acute lymphoblastic leukemia (ALL) cells, STF-083010 also reduced XBP1s levels in a dose-dependent fashion. HNA demonstrates similar effects to STF-083010 on the proliferation and survival of patient-derived pre-B ALL cells, and both inhibitors employ mechanisms that encompass cell cycle arrest at the G0/G1 phase. In addition, HNA significantly increased survival in xenotransplant-recipient mice, injected with pre-B ALL cells at lower cell counts (50,000 and 10,000). Besides pre-B ALL cells, the inhibitory molecules managed to affect Ph+ ALL cells, especially the ones carrying the Tyrosine Kinase Inhibitor (TKI)-resistant mutant BCR-ABL1^T315I^, whereas mature B-cell lymphoma and MM cells were significantly less sensitive to treatment [123]. The antileukemic activity of STF-083010 was also confirmed in human MM xenograft model and freshly isolated CD138+ human MM cells [125]. Furthermore, STF-083010 was demonstrated to possess fungistatic properties, as it inhibited colony growth of cultured *A. fumigatus*, whereas Toyocamycin did not [126]. Alike 4µ8C, STF-083010 reduced lipid-induced metainflammation and alleviated progression of atherosclerosis upon administration to macrophages and ApoE^−/−^ mice on a Western-type diet [31].

#### 4.3.4. Toyocamycin

Toyocamycin is an agent derived from *Actinomycete* strains that was shown to selectively inhibit ER stress-mediated cleavage of XBP1 mRNA in HeLa cells without affecting IRE1 phosphorylation. Moreover, toyocamycin suppressed the constitutive activation of XBP1 expression in MM cell lines, inhibited tumor growth in a MM xenograft in vivo model, and synergistically enhanced the chemotherapeutic effect of bortezomib in MM cells, including the bortezomib-resistant ones [127]. In multiple pancreatic cancer cell lines, toyocamycin decreased the clonogenic growth in a dose-dependent manner, exerted synergistic effect with bortezomib, 17-DMAG, gemcitabine or dasatinib, and affected the mitochondrial membrane potential (MMP) of treated cells [117]. Toyocamycin reduced the viability of prostate cancer PC-3 cells via induction of apoptosis, mitochondrial dysfunction, ROS generation, and activation of MAPK-dependent signaling; however, it did not affect non-malignant RWPE-1 cells [128]. The inhibitor also abolished the clonogenic growth of human renal cell carcinoma (RCC) under hypoxic conditions, and reduced cyclosporine A-induced expression of VEGF and IRE1 activation, which is of particular therapeutic interest in the context of organ transplantation [129].

#### 4.3.5. MKC Inhibitors

One representative of the MKC compounds series is the inhibitor MKC-4485. Administration of MKC-4485 reduced IRE1α activity and decreased levels of XBP1s in MODE-K cells. The supernatant from MKC-4485-treated MODE-K cells triggers activation of dendritic cells. Interestingly, the results of this study suggest that reduced IRE1α activity plays a crucial role in the activation of both dendritic and T cells, which is responsible for the antitumor immunity observed in Rnf5^−/−^ mice [130]. However, in Exn5/Exn5 HCT116 cells, administration of MKC-4485 reduced XBP1s levels but did not protect the cells against ER stress-induced death; this indicates that IRE1-dependent signaling does not contribute to the resistance of the HCT116 Exn5/Exn5 phenotype [131].

On the other hand, MKC-8866 showed effectiveness against GBM when combined with Stupp-like protocol (irradiation/TMZ treatment) in vivo. As the inhibitor does not pass the BBB, it was delivered intraoperatively by means of a biomaterial scaffold. Such combined treatment significantly increased mouse survival (~20%) compared to the Stupp protocol alone and resulted in a completely different tumor phenotype [132]. Co-administration of MKC-8866 with another chemotherapeutic agent, paclitaxel, enhanced tumor suppression and delayed tumor relapse after therapy in a triple-negative breast cancer (TNBC) xenograft mouse model [133]. Dual therapy with MKC-8866 and nilotinib showed a striking synergism against BCR-ABL1+ ALL cell lines, SUP-B15 and TOM-1, and the pro-apoptotic mechanism involved activation of p38 MAPK [134]. In prostate cancer (PC) cells, not only did MKC-8866 demonstrate synergistic antitumor activity with currently used chemotherapeutics, but also proved to be effective in monotherapy in PC murine models [135]. Intriguingly, treatment of rhabdomyosarcoma (RMS) cell lines with MKC-8866 significantly diminished cell viability, proliferation, and colony formation, with the alveolar RMS subtype being highly sensitive to MKC-8866 exposure, and the embryonal subtype susceptible to the selective PERK inhibitor AMGEN44 [136].

As in the case of 4μ8C, the MKC-3946 mutant abolished proinsulin-induced XBP1 mRNA splicing in insulinoma cell line [121]. In AML cell lines and patient samples, MKC-3946 also effectively blocked cleavage of XBP1 mRNA and exerted a cytotoxic effect, in a manner similar to that of STF-083010, HNA, and toyocamycin [124]. Its antineoplastic potency has also been confirmed in MM in vitro and in vivo; in these, the inhibitor triggered modest growth inhibition but did not affect normal mononuclear cells. In addition, MKC-3946 fostered bortezomib- and 17-AAG-induced ER stress and the resulting apoptotic cell death, and the effect was independent of the presence of bone marrow stromal cells or IL-6 [137].

#### 4.3.6. HAA

Apart from 4μ8C and MKC-3946, other aromatic ring systems containing hydroxy-aldehyde moieties that belong to the hydroxy–aryl–aldehydes (HAA) class have recently been identified [138]. HAA inhibitors have been demonstrated to selectively target IRE1α RNase function, and thus inhibit both XBP1 splicing and RIDD activity in the cellular context. Similar to other direct RNase inhibitors, the mechanism of action of HAA involves Schiff base formation with the K907 residue. Additionally, HAA molecules dock into a shallow pocket at the RNase-active site, which involves pi-stacking interactions with H910, F889, and Y892 [139]. A detailed understanding of present molecular interactions may lead to development of novel HAA-derivative IRE1 inhibitory compounds that could have potential clinical use in the future.

#### 4.3.7. B-I09

B-I09 is a novel, highly selective RNase inhibitor with a structure based on tricyclic chromenone. It was recently developed to improve the cellular and in vivo efficacy, bioavailability, and pharmacokinetics of the existing inhibitors. B-I09 potently suppressed XBP1 activity and triggered apoptosis in CLL cells, and effectively attenuated leukemic progression in CLL-bearing Eμ-TCL1 mice, without inducing systemic toxicity. Mechanistically, B-I09 compromised B cell receptor (BCR)-dependent signaling in treated cells but did not affect the secretory and integral membrane-bound protein transport. Upon co-administration with the BTK inhibitor ibrutinib, the two drugs synergize to orchestrate apoptosis in several hematopoietic malignancy models, including B cell leukemia, lymphoma, and MM [140]. Additionally, B-I09 has recently been found to exert synergistic effects with doxorubicin against c-Myc-overexpressing Burkitt’s lymphoma (BL) [141]. Thus, further studies should examine whether the other tumors known to overexpress c-Myc, such as hepatocellular carcinoma, neuroblastoma, or PC [142], are equally sensitive to pharmacological modulation of IRE1 signaling.

#### 4.3.8. K114

Recently, a novel, luciferase reporter-based in vivo assay using medaka fish was developed for more detailed investigation of IRE1 inhibitors and monitoring of their pharmacokinetics. This phenotypic screening approach identified K114 from 1280 other compounds. The inhibitor proved to be effective against ER stress-induced XBP1 mRNA splicing in human colorectal cancer HCT116 cells, and it was shown to inhibit the cleavage of XBP1 mRNA by purified IRE1-C in vitro as effectively as 4μ8C, in a dose-dependent manner. In the reported assay, usage of the investigated inhibitor appeared to be detrimental to ATF6-knockout medaka, as it induced intracerebral hemorrhage in ATF6α^−/−^ embryos, but not in the wild-type ones. Interestingly, the effective concentration of 4μ8C in the mentioned in vivo model was significantly higher than the one established in vitro. This phenomenon was presumably associated with the formation of a Schiff base with K907, which makes 4μ8C prone to nonspecific inactivation in vivo [29].

### 4.4. Other Inhibitors Specific for IRE1

Several acridine derivatives have recently been identified using a luciferase reporter–based HTS and topological data analysis (TDA), among which the N^9^-(3-(dimethylamino)propyl)-N^3^,N^3^,N^6^,N^6^-tetramethylacridine-3,6,9-triamine (3,6-DMAD) demonstrated the most potent inhibition of IRE1-dependent signaling. 3,6-DMAD disrupted oligomerization of IRE1 and inhibited RNase activity with the resulting XBP1 mRNA splicing in the novel, unique mechanism of action, distinct from that of any other existing IRE1 inhibitor. Moreover, 3,6-DMAD exerted a cytotoxic effect in MM cell lines and suppressed the growth of MM tumor xenografts in vivo via inhibition of IRE1/XBPs signaling [138]. These findings provide a strong preclinical rationale for potential application of 3,6-DMAM in future therapeutic approaches against MM. Nevertheless, further studies including SAR analysis are required in order to clarify the detailed structural properties of 3,6-DMAD and the mechanisms underlying its inhibitory effect.

### 4.5. IRE1 Activators

#### 4.5.1. Quercetin

The flavonol quercetin was demonstrated to activate IRE1 RNase function in yeast. As revealed by structural analysis, the compound potentiates ADP-mediated activation of IRE, with the engagement of a nucleotide-binding cleft at the dimer interface of the RNase domain, distinct from the previously described nucleotide-binding site. Therefore, it has been suggested that the mechanism of action of quercetin involves enhanced dimer formation. These findings shed light on the novel possibilities of potential modulation of IRE1-dependent signaling and may assist in the development of further modulatory compounds interacting with mentioned unanticipated site of the RNase domain [143].

#### 4.5.2. NM-PP1

1NM-PP1 is an ATP mimetic that has been demonstrated to activate RNase by an allosteric mechanism by binding specifically to a designed pocket of an IRE1(I642G) mutant with a disabled kinase domain. This provided evidence that the requirement for ATP binding can be bypassed by incubation with 1NM-PP1, and that the activation of kinase domain can be entirely bypassed for induction of RNase activity [144,145]. Notably, upon application of the 1NM-PP1 in IRE1(I642G) overexpressing cells, XBP1 mRNA splicing was induced, even in the absence of ER stress, whereas RIDD was not [93]. Surprisingly, upon development of IRE1 kinase domain mutants particularly sensitized to the effects of 1NM-PP1, it has been found that obtained mutants were not inhibited by 1NM-PP1, but, conversely, required the drug as a cofactor for their activation. These findings suggest that it is ATP competitive ligand-induced conformational change that is responsible for the activation of the kinase domain and its associated molecular events, not necessarily the phosphotransfer reaction [144].

#### 4.5.3. Other Activators of IRE1 Function

In contrast to the allosteric activators of RNase domain described above, recent work has identified a new class of ATP-competitive RNase activators displaying high selectivity and strong cellular activity. The two most potent allosteric IRE1 activators identified so far, namely G-9807 and G-1749, were selected by means of ATP-competitive TR-FRET assay. Both IRE1 modulators bound to the kinase domain and induced RNase activation with low-nanomolar IC50 values. Treatment of the KMS-11 MM cell line with G-9807 and G-1749 resulted in significant elevation of the XBP1s mRNA cellular levels and decreased levels of the RIDD-specific target, DGAT2. One of the selected molecules, G-1749, proved to be potent and to demonstrate nigh kinase selectivity towards IRE1, and neither G-9807 nor G-1749 inhibited PERK. Moreover, it has been found that G-1749 is structurally similar to the C_31_H_29_ClN_6_O_3_S (KIRA8); however, the two molecules exert opposing effects on the modulation of RNase activity. G-1749 promotes IRE1 RNase activity via occupation of the kinase pocket proximal to the DFG motif, which leads to a distinct conformation of the activation loop (DFG-in or C-helix in), and this mechanism does not stabilize the dimer interface [146]. These finding suggests that the IRE1 kinase activation loop may play a role in the regulation of RNase activity.

IXA1, IXA4, and IXA6 are novel, non-toxic molecules capable of inducing IRE1 RNase activity; all were identified through HTS approach. All molecules were found to selectively activate IRE1/XBP1s signaling. This mechanism was highly specific, independent of binding the ATP-binding pocket, and did not globally activate other signaling pathways related to ER stress, including heat shock response and oxidative stress response. Interestingly, exposure of HEK293T cells to IXA4 evoked XBP1 splicing, but not the other IRE1-specific activities such as RIDD or JNK phosphorylation, which are associated with the activation of IRE1-dependent signaling under chronic stress conditions. In SH-SY5Y cells expressing amyloid precursor protein (APP) mutants, that are directly associated with the pathogenesis Alzheimer’s disease (AD), IXA4 increased targeting of APP to degradation in IRE1-dependent fashion, and thus contributed to reduced Aβ secretion and improvement of ER proteostasis. Furthermore, IXA4 alleviated mitochondrial toxicity related to overexpression of mutant APP [147]. These findings emphasize the unique character of the investigated modulatory compounds as compared to previously described IRE1 activators. Nevertheless, further studies are needed in order to assess their potency in the other experimental models and under varying conditions.

## 5. Summary and Perspective

As the most conserved and the most well studied branch of the UPR, IRE1 has been suggested to be a key player in the regulation of intracellular homeostasis and cell fate determination. It is also for these reasons that IRE1 is considered as technically easier to target by pharmaceuticals, as compared to PERK or ATF6. Recent structural analyses have provided a deeper insight into molecular mechanisms underlying the activation of IRE1 and of its corresponding, downstream signaling pathways. On the contrary, the initiating mechanism and regulatory components associated with activation of the molecule remain much more elusive and controversial, with the multiple models being proposed. As described above, IRE1 can become activated in distinct ways: Via dissociation of BiP, direct interaction with misfolded protein or lipid disequilibrium, and the activity is regulated by numerous factors like ERdj4, RNH1, or XBP1u, but more detailed characteristics of regulatory events that fine tune IRE1-dependent signaling are yet to be established. The unique, separate activity of the two enzymatic domains of IRE1, which can be targeted independently of each other, makes it a desirable feature for experimental evaluation of distinct IRE1 functionalities and development of potential pharmacological approaches. Importantly, the ability of IRE1 to induce apoptotic cell death via TRAF2/JNK cascade, without the involvement of RNase domain, may serve as another interesting therapeutic target. The IRE1-driven cell fate determination is also dependent on the establishment of the balance between XBP1 splicing and RIDD activities. According to one theory, it is the oligomerization of IRE1 molecules into higher-order assemblies that drives the maximal splicing activity. All these molecular events associated with the activation and signaling of IRE1 are crucial for establishing its role in human pathologies and development of new therapeutics.

In view of the newest findings, there is no doubt that it is the timing of the stressful stimuli that eventually switches the IRE1 response from pro-survival towards pro-apoptotic and results in execution of apoptotic cell death. Accordingly, ER stress may in fact be overcome with gradually switching of the respective UPR signaling pathways, from ATF6 towards IRE1/XBP1, whereas at the late phases, the cell fate depends on inactivation of IRE1 with a persistent, pro-apoptotic PERK activity. Of note, the IRE1 branch has been demonstrated to interact with other signaling pathways, like MAPK and mTOR, as well as with the other two arms of the UPR. In particular, the essential role of ATF6 signaling in maintenance of IRE1-dependent XBP1s processing should be taken into account. The fact that both IRE1- and PERK-dependent pathways converge on CHOP-mediated DR5 processing with the subsequent induction of pro-apoptotic signaling indicates for importance of integration between these UPR branches in determining cell fate under stressful conditions. Further studies are needed to distinguish the separate activities of the two molecules.

As a plethora of cellular functions is under control of the UPR signaling cascades, these could be targeted in various contexts of human diseases. Basal UPR signaling is pivotal for proper functioning of secretory cells like plasma B cells, which require tight regulation of secretory mechanisms and are prone to protein overload. In particular, XBP1 has been demonstrated to regulate the synthesis of immunoglobulins as well as expression of genes encoding for early B-cell antigens. Conversely, persistent overactivation of this pathway under chronic stress conditions is linked to numerous pathologies and was shown to promote survival of certain cancer types. With this regard, emerging evidence suggests the potential of pharmacological inhibition of IRE1 signaling in the case of highly secretory malignancies with high utilization of XBP1s signaling, like insulinoma or multiple myeloma. Studies summarized here support this concept, as a broad range of specific IRE1 inhibitors were tested with promising results in preclinical MM in vitro and in vivo models, as well as in numerous leukemic and pancreatic cancer cell lines. Importantly, KIRA8 and several RNase inhibitors (HNA, STF-083010, MKC-8866, and MKC-3946) did not alter functioning of nonmalignant cells and were well tolerated in in vivo studies. Furthermore, the fact that leukemic cells benefit from UPR signaling to acquire drug resistance provides a strong rationale for use of IRE1 inhibitors in combined therapeutic approaches; this is of utmost importance especially in terms of resistance to TKIs. Consistently, the effectiveness of STF-083010 and HNA against the Ph+ ALL cells with TKI-resistant BCR-ABL1^T315I^ mutation is indicative of their great potential for novel, personalized molecular therapy against this type of leukemia. The other aspect of IRE1-targeted antitumor therapy that could be assessed is the expression of pro-angiogenic factor VEGF, the regulation of which was also shown to depend on IRE1 signaling.

Understanding the crosstalk between the complex intramolecular signaling pathways and the way it executes cell fate decisions may lead towards discovery of novel therapeutic approaches as regards selective IRE1 inhibitors. Nevertheless, it should be kept in mind that selective inhibition of the respective kinase and RNase activities of IRE1 leads to differing effects, which is strictly associated with the binding site that the modulatory compounds interact with and the potential conformational changes of the enzyme. Such pharmacological intervention may also involve some compensatory mechanisms within the other branches of the UPR, which requires further investigation. The effects of IRE1 inhibition by specific molecules should be therefore investigated carefully, with special regard to their potential off-target properties, including affinity for other kinases. Although kinase inhibitors provide a unique opportunity for modulation of RNase activity of IRE1 without interaction with RNase domain, direct RNase inhibitors seem to be more favorable candidates for application in preclinical models. As a complete inhibition or activation of the specific UPR signaling pathways may in fact cause severe on-target toxicity, and this effect is not desirable in non-cancer cells, it is warranted to exploit the partial modulators of IRE1 signaling, that can provide a sort of restoration of the UPR function or modulate it in a unique way. Another problem to be solved is the actual mechanism of action of several newly discovered inhibitors like 3,6-DMAD, which could not be classified either as type I or type II kinase inhibitor.

A plethora of studies indicate that loss of IRE1/XBP1 signaling significantly impairs cell survival, which is of particular importance in the context of neurodegenerative diseases, infarction, and several metabolic disorders. In the light of this latest evidence for the cytoprotective role of IRE1 suggesting that it is in fact the abolishment of IRE1 signaling that contributes to cell apoptosis, the development of specific IRE1 activators and extensive evaluation of their properties should also be considered. Intriguingly, one of such molecules, G-1749, potently activate RNase domain in contrast to KIRA8 inhibitor, despite structural similarity of the two compounds and the same binding site; this finding reflects the importance of further structural studies on the regulatory sites within kinase domain. The other newly developed IRE1 activators (IXA compounds) induce RNase activity in a highly specific manner, distinct from that of type I kinase inhibitors, and modulate IRE1/XBP1s signaling to moderate levels (~30–40%), which considerably reduces the risk of potential on-target toxic effects. The careful evaluation of multiple cellular and animal models of distinct genetic and physiological backgrounds is crucial for more refined assessment of the efficacy of novel IRE1 modulators, especially in the context of innovative, reporter-based in vivo assays. Along these lines, further research is needed to answer all unresolved issues, continue the development of IRE1 modulatory compounds, and eventually translate them into clinical practice.
